# Bacterial cell identification in differential interference contrast microscopy images

**DOI:** 10.1186/1471-2105-14-134

**Published:** 2013-04-23

**Authors:** Boguslaw Obara, Mark AJ Roberts, Judith P Armitage, Vicente Grau

**Affiliations:** 1 School of Engineering and Computing Sciences, University of Durham, Durham, UK; 2 Oxford Centre for Integrative Systems Biology, University of Oxford, Oxford, UK; 3 Department of Biochemistry, University of Oxford, Oxford, UK; 4 Oxford e-Research Centre, University of Oxford, Oxford, UK; 5 Institute of Biomedical Engineering, University of Oxford, Oxford, UK

## Abstract

**Background:**

Microscopy image segmentation lays the foundation for shape analysis, motion tracking, and classification of biological objects. Despite its importance, automated segmentation remains challenging for several widely used non-fluorescence, interference-based microscopy imaging modalities. For example in differential interference contrast microscopy which plays an important role in modern bacterial cell biology. Therefore, new revolutions in the field require the development of tools, technologies and work-flows to extract and exploit information from interference-based imaging data so as to achieve new fundamental biological insights and understanding.

**Results:**

We have developed and evaluated a high-throughput image analysis and processing approach to detect and characterize bacterial cells and chemotaxis proteins. Its performance was evaluated using differential interference contrast and fluorescence microscopy images of *Rhodobacter sphaeroides*.

**Conclusions:**

Results demonstrate that the proposed approach provides a fast and robust method for detection and analysis of spatial relationship between bacterial cells and their chemotaxis proteins.

## Background

Modern bacterial cell biology has been revolutionised with the use of fluorescent markers coupled with microscopy allowing the visualisation of sub-cellular localisation in the bacterial cell. Generating contrast in these images to determine the cell boundary is achieved using a number of optical methods including Phase Contrast and Differential Interference Contrast (DIC) microscopy both of which depend on light changing its properties as it passes through the sample.

It is increasingly important to interpret microscopy images in a quantitative manner thus being able to reconstruct the cell boundary from these images is of great importance, allowing the location of fluorescent markers to be determined within the bacterial cell. In particular this information can allow correlation of the position of proteins within the bacterial cell throughout the cell cycle.

Phase contrast images provide a clear light dark boundary around the entire cell making it easy to determine by computational methods [1]. Software has been developed to analyse phase contrast fluorescent microscopy images [2] however this software does not enable the analysis of DIC images. DIC microscopy has the advantage of no phase ring in the objective and a larger depth of field making it advantageous for fluorescence microscopy in bacterial cell biology where the signals are often from a small number of fluorphores. It also enables better time courses to be taken with less photobleaching with less illumination needed per excitation. A lower excitation time per image results in a reduced level of photobleaching which is dependent on the intensity and duration of the excitation light. However in DIC image the cell outline is much more difficult to extract being made of both light and dark regions. In this paper we describe a new method for reconstructing the cell boundary from a DIC image. This then allows quantisation of the fluorescence image.

### DIC imaging

**Figure 1:**
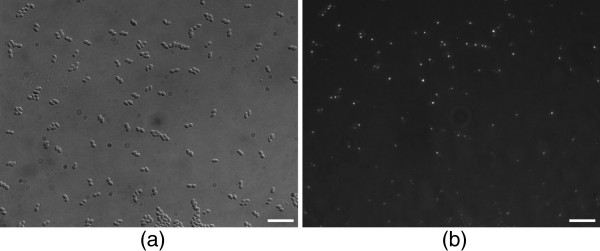
**DIC image of bacterial cells and chemotaxis proteins.(a)** DIC image of bacterial cells. **(b)** Fluorescent image of chemotaxis proteins. Scale bars correspond to 10 [*μm*].

The width of the emission spectrum of a common fluorophore allows only for a limited number of spectrally distinct fluorescent markers in the visible spectrum, which is also the regime where CCD-cameras are used in microscopy. For imaging of cells or tissues, it is necessary to obtain an image from which the morphology of the whole cell can be extracted. This can be achieved by differential interference contrast microscopy [1]. However, typically, DIC images appear with a bas-relief profile caused by the gradient of the optical path length and a phase shift between the two beams. For the human eye these DIC images are easy to interpret, see Figure 1. However, automatic image analysis of DIC scans with hundreds of cells of different shapes and partially weakly identifiable contours is difficult. In the direction of the DIC shear vector, the intensity distribution of the cell is characterized by a transition of bright to dark, resulting in a well-defined contrast. But in the perpendicular direction to the DIC shear there is no contrast against the background, and hence a lack of information about the complete cell boundary.

### DIC image segmentation

Standard image processing methods for DIC image segmentation such as thresholding or edge detection produce insufficient results [3-5], with discontinuous regions or edges and can only be used under additional assumptions for the shape or by template constructions [6]. Moreover, the use of deformable templates has been proposed [7,8]. These are modelled closed curves, which are fitted to object boundaries in iterative processes. More recently, [9] has demonstrated that the combined analysis of local image entropy and local illumination intensity could be suitable to identify individual cells with high sensitivity and specificity at low computational cost. However, all of these techniques are either limited to cell types with relatively constant sizes and shapes, or require relatively long processing times, making them unsuitable for high-throughput applications. The alternative to these techniques is to perform DIC image reconstruction and then apply much simpler and more robust segmentation techniques.

### DIC reconstruction

[10] has presented an excellent description, discussion, comparison and critical evaluation of most existing DIC image reconstruction methods. Most common iterative solutions are based on line integration and deconvolution [11], variance filtering and directional integration using iterative energy minimization [12], and rotational diversity [13]. The latter technique involves taking several rotated DIC images and combining them using iterative deconvolution. Non-iterative methods include direct deconvolution [14] and the half-plane Hilbert transform [15] which is a qualitative Fourier-space approach to integrating the phase gradient. Moreover, a combination of the Hilbert transform and line integration method has been explored by [10].

Here we propose a high-throughput bioimage informatics approach to detect and characterize complex bacterial cells and chemotaxis proteins. The developed approach is based on a DIC shear orientation detection, followed by DIC image reconstruction and local segmentation of bacterial cells and chemotaxis proteins. Finally, an analysis of spatial relation between bacterial cells and their chemotaxis proteins is performed. *R. sphaeroides* was chosen because this is a small bacterial cell, generally 2 [*μm*] long and has chemosensory proteins known to localise in both the cytoplasm and the membrane. It has also previously been shown that there is cell cycle dependent positioning of chemotaxis [16] proteins making this an ideal test for this software. The small size mean that automated analysis is important to reduce potential error in human measurement.

## Results

### Validation of the cell segmentation procedures

#### *DIC shear direction estimation*

**Figure 2:**
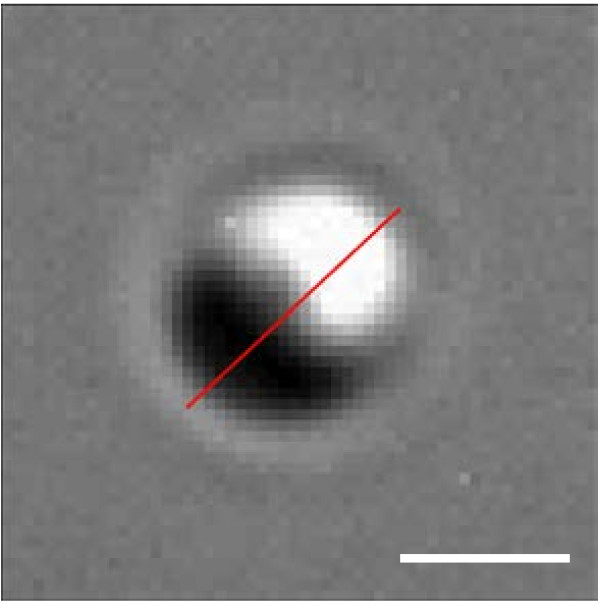
**DIC shear direction estimation procedure.** DIC shear direction estimation from image of spherical bead of diameter 1 [*μm*]. An estimated orientation *a*_*μ*_ = 43.52 [deg] (red line) was calculated with a rotated linear structuring element defined by *l* = 20 [pixels] and *n* = 180. Scale bar corresponds to 1 [*μm*].

We tested the performance of the proposed approach for DIC shear direction estimation on images of synthetic spherical beads. An illustration of such an analysis is demonstrated in Figure 2.

#### *DIC image reconstruction*

We have applied the DIC reconstruction approach in a challenging bioimaging application, based on the description in Methods Section, to extract and analyse bacterial cells and chemotaxis proteins in images of *Rhodobacter sphaeroides*.

To quantify the performance of the DIC image reconstruction approach, seven complex images were selected. Manual segmentation of 290 bacterial cells was performed by an expert using the Pencil tool in Adobe Photoshop [17], as *ground truth* (*C*_*GT*_). *C*_*GT*_ cells and cells detected by the proposed approach (*C*_*A*_) were compared using the Dice coefficient [18]. In our case, the Dice similarity coefficient is defined to find the overlapped regions between *C*_*GT*_ and *C*_*A*_ cell bodies in an image. The Dice coefficient is given by:

(1) $$D=\frac{2|C_{\text{GT}}\cap C_{A}|}{\left|C_{\text{GT}}|+|C_{A}\right|}$$

**Figure 3:**
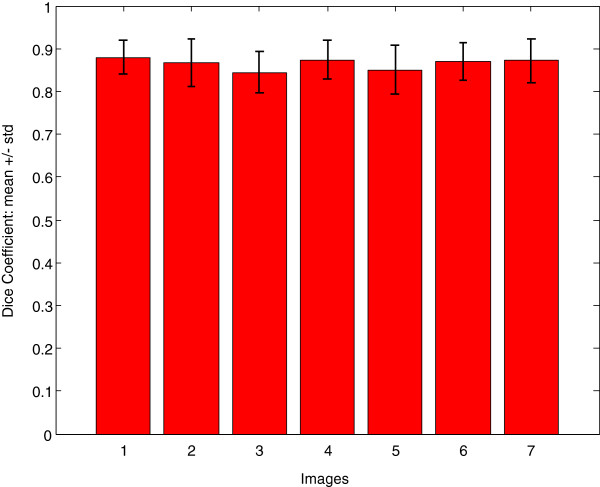
**Validation - dice coefficient.** Comparison of computational vs. manual method for 7 images. Bar plots show average Dice coefficients and error bars indicate one standard deviation.

*D* lies between 0 to 1. If *C*_*GT*_ = *C*_*A*_, then *D* = 1.0 (perfect overlap), and if *C*_*GT*_ does not overlap with *C*_*A*_, then *D* = 0 (no overlap). Comparison of overall accuracy for all analysed images is presented in Figure 3. The Figure shows, for every image, an average Dice coefficient value and error bar indicating its standard deviation. For all 290 analysed cells, the Dice coefficient average value was *D* = 0.8635±0.0513, while values of *D* = 0.8043±0.1096 were obtained when comparing two manual segmentations performed at different times by the same expert of the same image set.

In addition to the *Rhodobacter sphaeroides*, a set of 30 polystyrene spherical beads (PolySciences Inc) was used to validate the performance of the DIC image reconstruction approach. Each synthetic bead had a diameter of 1 [*μm*] and an area of 0.7854 [*μm*^2^]. For all reconstructed beads, the area average value was 0.8007 ±0.0312 [*μm*^2^].

**Figure 4:**
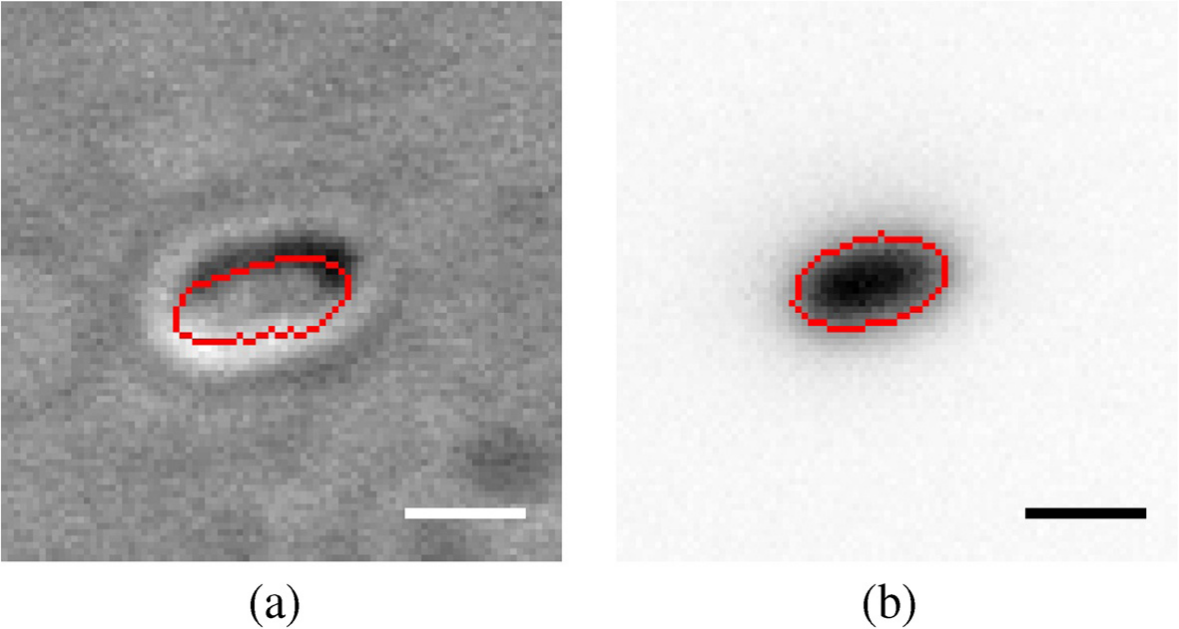
**Validation - DIC vs. fluorescence.** Comparison between segmentations of bacterial cell observed on DIC **(a)** and fluorescence **(b)** microscopy images. An overlay of the segmented cell body is in red. Scale bars correspond to 1 [*μm*].

Furthermore, to compare the relationships between actual bacterial cell body and the same cell body determined with a use of the DIC microscopy, a set of 20 bacterial cells have been stained and then imaged using fluorescence and DIC microscopy, see Figure 4. The stained bacterial cells, observed on fluorescence images, were segmented using the standard thresholding method described in the Image segmentation Section. The same bacterial cells, observed on DIC images, were reconstructed and segmented using the automated DIC image reconstruction approach. For all analysed cells, the area average values were 1.4748±0.2785 [*μm*^2^] and 1.4737±0.2396 [*μm*^2^], for bacterial cells on fluorescence and DIC images respectively.

### Spatial relations

**Figure 5:**
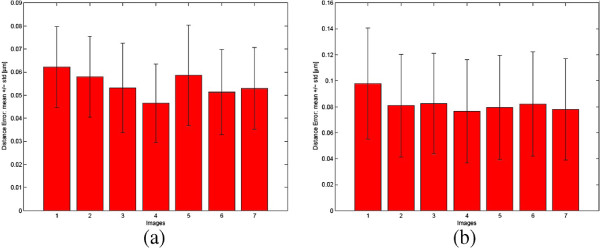
**Validation: distance error.** Comparison of computational vs. manual method for 7 images. Bar plots show average distance errors *ϵ*_*d*_ and error bars indicate one standard deviation, for centroids **(a)** and centrelines **(b)** respectively.

The proposed procedure for the analysis of the spatial relations between the cell and its chemotaxis proteins, as described in the Image analysis Section, has been also reviewed on the same set of test images containing 290 cells. To quantify the performance of the proposed approach for analysis of the spatial relations between the cell and its chemotaxis proteins, estimated centroids and centrelines, for *C*_*GT*_ and *C*_*A*_ cells, were compared using the distance error *ϵ*_*d*_ measure proposed in [19]. In case of a centreline, the distance error is defined as the average distance between each point on the centreline of *C*_*GT*_ and the corresponding closest point on the centreline of *C*_*A*_. Distance error analysis of the cell centroids and centrelines, for all analysed images, is presented in Figures 5(a) and 5(b). For all 290 analysed cells, the distance error average values were *ϵ*_*d*_ = 0.0551±0.0192 [*μm*] and *ϵ*_*d*_ = 0.0818±0.0402 [*μm*], for centroids and centrelines respectively.

**Figure 6:**
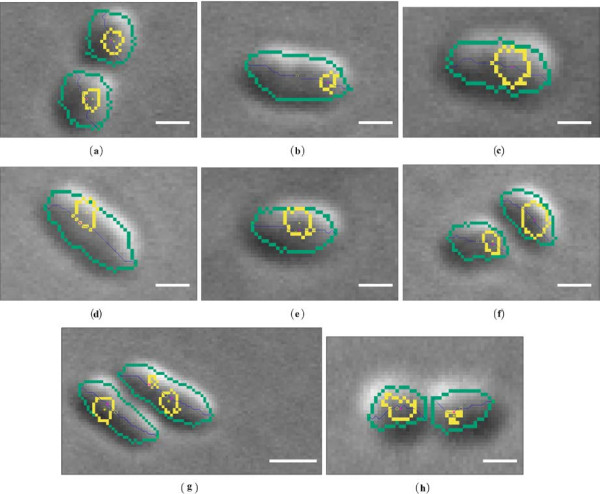
**Results of the proposed approach for the analysis of the spatial relations between the cell and its chemotaxis proteins (a-h).** Legend: (green) - cell boundary, (yellow) - chemotaxis protein boundary, (yellow circle) - cell centroid position (*r*), (yellow star) - chemotaxis protein position (*c*_*i*_), (magenta star) - point on the centreline $p_{c_{i}}$ which correspond to *c*_*i*_, (green cross) - arbitrary end of the cell. Scale bars correspond to 1 [*μm*].

Additionally, obtained results of the analysis of the spatial relations between the cell and its chemotaxis proteins, for a set of selected cells, is presented in Figure 6.

## Discussion

An image processing and analysis concept for general differential interference contrast microscopy image segmentation has been developed (see DIC images: bacterial cells segmentation Section), based on the DIC image reconstruction method using the 2D Hilbert transform with a direction of reference in the Fourier domain.

**Figure 7:**
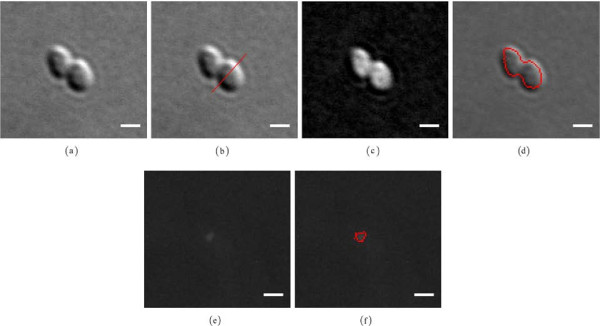
**Bacterial cell and chemotaxis protein body segmentation.** Bacterial cell and chemotaxis protein body segmentation workflow: **(a)** DIC input image, **(b)** DIC shear direction estimation: an obtained orientation *a*_*μ*_ = 48.20 [deg] (red line) calculated by use of a rotated linear structuring defined by *l* = 20 [pixels] and *n* = 180, **(c)** DIC image reconstruction, **(d)** an overlay of the segmented cell body in red, **(e)** fluorescent input image, **(f)** an overlay of the segmented chemotaxis protein body in red. Scale bars correspond to 1 [*μm*].

To evaluate the performance of the proposed approach, we applied it to images of the small bacterium *Rhodobacter sphaeroides* with fluorescently tagged chemosensory proteins, and the results are presented in Results Section. Visual inspection of the results confirm the robustness of the proposed approach for bacterial cells extraction and analysis of spatial relationship between bacterial cells and their chemotaxis proteins (see Figures 7 and 6). Furthermore, we compared quantitatively the results obtained by the proposed approach with the *ground truth* results, delineated manually by an expert. As shown in Figure 3, the performed evaluation, based on the Dice’s similarity coefficient measure, demonstrates the accuracy and effectiveness of the proposed approach. Additionally, to address an issue of the DIC imaging producing a relatively large boundary of an imaged object, the proposed DIC image reconstruction approach was validated by comparing our values with ground truth measurements in synthetic beads.

This work lays the groundwork for using DIC to produce spatiotemoral maps of proteins within the bacterial cell cycle. The use of DIC gives the potential for working with smaller levels of photobleaching and generating higher resolution maps for bacterial cell biology. This new method allows the application of high throughput analysis of low copy number bacterial proteins throughout the cell cycle. The multiple parameters measured allows the determination of how any movement or positioning varies with the cell cycle and age of cell.

Future work will be focused on high-throughput measurements of the *Rhodobacter sphaeroides* observed on temporal sequences of images. We will also investigate the applicability of the proposed concept to detect other bacterial cells observed in DIC images.

## Materials

### *Rhodobacter sphaeroides*

*R. sphaeroides* strains were grown aerobically in succinate medium [20] at 30°C with shaking at 225 rpm. Mid log phase cells were immobilized on a thin layer of 0.8% agarose in succinate medium on microscope slides [21].

One strain JPA1558 (TlpT-YFP) was used [21] enabling the visualisation of the cytoplasmic chemotaxis clusters using fluorescent microscopy. TlpT is used as a marker for the cytoplasmic cluster and CheA2 for the membrane cluster.

### Imaging

DIC microscopy and fluorescence images were acquired with a Nikon TE200 microscope and YFP filter set (Chroma, Rockingham, VT) and recorded with a cooled charge-coupled device camera (Hamamatsu Photonics, Hamamatsu City, Japan). A Nikon oil immersion 100x objective was used with an ND of 1.49. The final resolution achieved is 0.065573*x*0.065573 [*μm*] per pixel DIC illumination was achieved using Nikon Eclipse TE200 DIC attachment. This uses two Wollaston prisms and two polarisers to generate DIC. The 2nd polariser is mounted in the emission filter wheel and thus is not present when a fluorescence image is being acquired. Each slide had up to three images taken and images were analysed from three independent days, to ensure any analysis methods was independent of slide position or any differences in slide preparation.

## Methods

**Figure 8:**
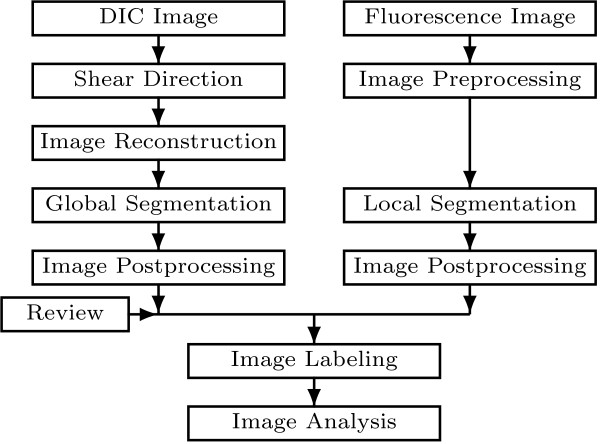
**The workflow approach.** The workflow of the proposed bioimage informatics approach for bacterial cells and their chemotaxis proteins identification in differential interference contrast and fluorescence microscopy images.

Here we introduce an image processing approach for high-throughput detection and characterization of complex bacterial cells and chemotaxis proteins as observed on DIC and fluorescence microscope images (see Figure 1). The workflow of the proposed approach is presented in Figure 8 and its execution is demonstrated in Figure 7.

For the DIC images, first, a robust DIC shear vector orientation detection procedure based on morphological gradient with linear structuring element and directional statistics are applied. Estimated orientation is then used by a DIC image reconstruction method where a 2D Hilbert transform with a direction of reference in the Fourier domain is employed. Global thresholding followed by a morphological filtering of the reconstructed image allows the detection of the bacterial cells. Additionally, a review process for ensuring quality of cells segmentation procedure is implemented.

For the fluorescence images, a combination of morphological filtering and local thresholding allows detection of the chemotaxis proteins.

Finally, an analysis of the spatial relationship between segmented cells and their chemotaxis proteins is performed.

### DIC images: bacterial cells segmentation

#### *DIC shear direction*

Let us consider an image *I*(**p**), where **p** = [*x*,*y*]^*T*^ is a column vector representation of the spatial location. For the detection of object boundaries, we use the morphological gradient of the image by a structuring element *S*[22], defined as the difference between the morphological dilation *δ* and erosion *ϵ* of the image by structuring element *S*:

(2) $$g_{S}(I)=\delta_{S}(I)-\epsilon_{S}(I)$$

where the dilation *δ* of an image *I* by a structuring element *S* is defined as a locus of points *z* such that *S* hits *I* when its origin coincides with *z*:

(3) $$\delta_{S}(I)=\{z:S_{z}\cap I\neq \emptyset \}$$

and the erosion *ϵ* is defined as follows:

(4) $$\epsilon_{S}(I)=\{z:S_{z}\subset I\}$$

Here we assume that the DIC shear direction, observed in DIC images (Figure 7(a)), can be estimated by the highest output of its morphological gradient *g* calculated using rotated linear structuring elements *S*_(*l*,*a*)_, defined by length *l* and direction angle *a*. Therefore, for each angle *a*_*n*_ defined as:

(5) $$a_{n}=\frac{180\cdot (n-1)}{N},\;\forall \;n\in \;[\;0,N]$$

where *N* is a number of directions, the morphological gradient of the image *I* is calculated and the sum of all its pixels is determined:

(6) $$w_{a_{n}}=\underset{p\in I}{\sum }g_{S_{\left(l,a_{n}\right)}}(I)$$

As can be noticed, a set $\left\{w_{a_{n}}\right\}$ has circular distribution properties, therefore, in order to estimate its maximum (which corresponds to its mean orientation *a*_*μ*_), directional statistics is applied. The estimation of *a*_*μ*_ is done by fitting a von Mises distribution function to the $\left\{w_{a_{n}}\right\}$ data using a multidimensional unconstrained non-linear minimization method [23]. In directional statistics, the von Mises distribution is a continuous probability distribution on the circle. This distribution is a circular analogue of the normal distribution and is defined as:

(7) $$f_{M}(\{w_{a_{n}}\}|a_{\mu },\kappa )=\frac{e_{\kappa \cos(a-a_{\mu })}}{2\pi B_{0}(\kappa )}$$

where the parameters *a*_*μ*_ and 1/*κ* are analogous to *μ* and *σ*^2^ (the mean and variance) in the normal distribution. *B*_0_ is the modified Bessel function of order zero.

**Figure 9:**
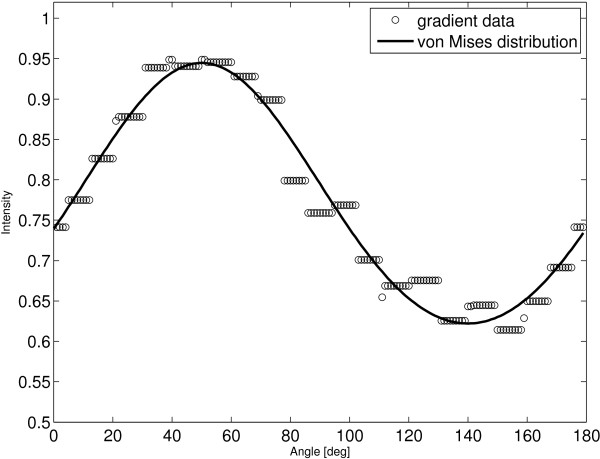
**DIC orientation estimation.** von Mises distribution fit to a gradient values computed from DIC image (Figure 7) by use of a rotated linear structuring defined by *l* = 20 [pixels] and *n* = 180. Estimated mean orientation is *a*_*μ*_ = 48.20 [deg].

The performance of this procedure applied to DIC image in Figure 7(a) is demonstrated in Figures 9 and 7(b).

#### *DIC reconstruction*

For a given DIC image *I* with DIC shear direction given by the orientation *a*_*μ*_, the DIC image reconstruction is computed by applying a directional Hilbert transform, as described in Multi-dimensional Hilbert transform Section. The DIC image is reconstructed as follows:

(8) $$I_{R}=ℜe(I_{{ℋ}_{ê}})$$

where $I_{{ℋ}_{ê}}$ is a Hilbert transform of the image *I* and its direction of reference in the Fourier domain is given by the unit vector defined as:

(9) $$ê\;=\;[\text{cos}(a_{\mu }),\text{sin}(a_{\mu })]$$

In order to reduce the side effects of the Hilbert transform, only positive part of the image *I*_*R*_ is considered:

(10) $$I_{F}=\left\{\begin{array}{cc} I_{R} & \;\text{if}\;I_{R}\geq 0\\ 0 & \;\text{if}\;I_{R}<0 \end{array}\right)$$

**Figure 10:**
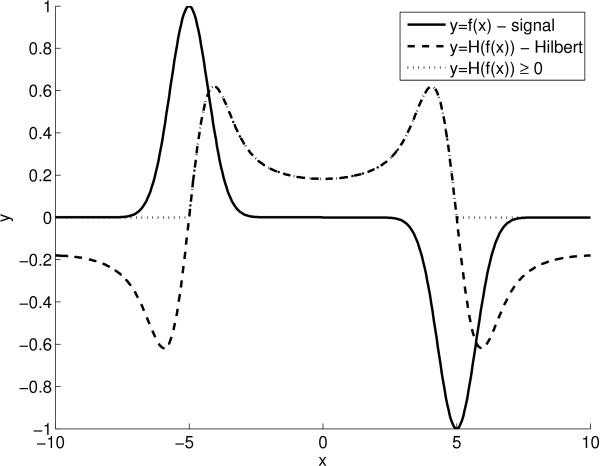
**DIC reconstruction approach.** The proposed approach for DIC reconstruction applied to 1D DIC-like signal.

The graphic representation of the above described procedure applied to 1D DIC-like signal, is presented in Figure 10. In this graph, the DIC-like signal (solid line) of a simple 1D object is modelled. In such a case, the DIC signal has the same levels inside and outside the object. Application of the Hilbert transform (dash line) to such a DIC signal, results in a signal which has a level increased inside and decreased outside of the object. A side effect of the Hilbert transform is the appearance of spurious signal minima on the external side of the object, which we eliminate by keeping only the positive values (dotted line) of the Hilbert transform.

Figure 7(c) shows the performance of the proposed DIC image reconstruction procedure.

#### *Image segmentation*

Application of the DIC image reconstruction allows the reconstructed image to be automatically analysed using global or local thresholding methods [24], which would not be applicable on the original DIC image. In our case, the image *I*_*R*_ is segmented by Otsu’s global thresholding method [25], see Figure 7(d).

#### *Image postprocessing*

Small elements in the segmented image *I*_*T*_ are removed by performing a morphological opening by reconstruction with a disk structuring element *S*_(*r*)_ of radius *r*:

(11) $$I_{G}=\rho_{S_{\left(r\right)}}(\gamma_{S_{\left(r\right)}}(I_{T}),I_{T})$$

where *γ*_*S*_ is an opening of an image *I* by a structuring element *S* defined as an erosion of *I* followed by a dilation with *S*:

(12) $$\gamma_{S}(I)=\delta_{S}(I)\;\epsilon_{S}^{T}(I)$$

*ρ*_*S*_(*I*,*J*) is defined as a morphological reconstruction of mask image *I* from marker image *J*, *J* ⊆ *I*, which is obtained by iterating geodesic dilation of *J* inside *I* until stability [22].

#### *Review*

Touching cells can be separated using watersheds [26], gradient flow [27] or active surfaces [28] method. In our case, a Euclidean distance map, calculated from touching cell boundaries, is used to calculate the watershed lines [29] which separates touching objects.

### Fluorescence images: chemotaxis proteins segmentation

#### *Image preprocessing*

In order to enhance low-contrast chemotaxis proteins within a fluorescence image *I*, the top-hat filtering *τ* with a disk structuring element *S*_(*r*)_ of radius *r* is applied:

(13) $$I_{H}=\tau_{S_{\left(r\right)}}(I)$$

#### *Image segmentation*

In order to segment the filtered image *I*_*H*_, a local thresholding method based on the mean of the local intensity distribution is applied. In our case, the local neighbourhood is represented by a circular window defined by a radius of 7 pixels. Resulting segmented image *I*_*K*_ is demonstrated in Figure 7(f).

#### *Image postprocessing*

Small elements in the segmented image *I*_*K*_ are removed by performing a morphological opening by reconstruction with a disk structuring element *S*_(*r*)_ of radius *r*:

(14) $$I_{O}=\rho_{S_{\left(r\right)}}(\gamma_{S_{\left(r\right)}}(I_{K}),I_{K})$$

### Image analysis

In order to analyse spatial relationships between bacterial cell and its chemotaxis proteins, the cell’s centreline has to be estimated.

#### *Cell centreline detection*

The most common approaches for centreline detection are based on Euclidean distance transform [30,31], fire propagation [32], Voronoi diagrams [33], clustering [34] and hybrid methods [35,36].

**Figure 11:**
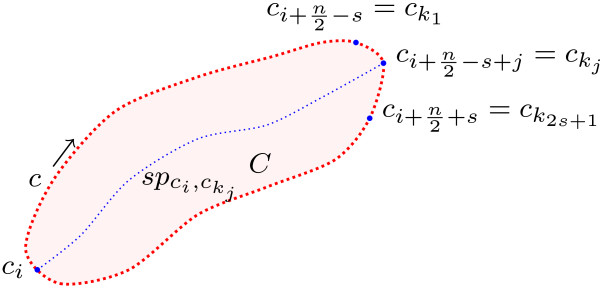
**Cell centreline detection procedure.** A schematic diagram showing the cell centreline detection procedure.

**Figure 12:**
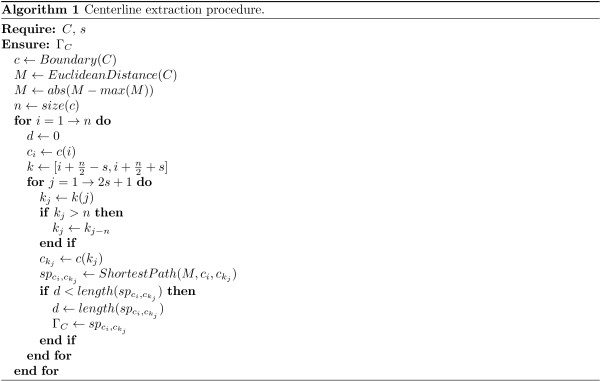
**Algorithm: centerline extraction procedure.** Centerline extraction procedure.

In our case, an approach which combines the Euclidean distance transform and the shortest path algorithm [37] is employed, and its workflow is shown in Figure 11 and Figure 12. In this approach, for every segmented cell *C* in the segmented image *I*_*G*_, the cell boundary *c* is extracted. Then, the Euclidean distance transform of the cell body is calculated to define a cost map *M*. Afterwards, for every point on the cell contour *c*_*i*_, the set of points *k* located near the half of the contour, in a range defined by *s*, is determined. Then, the shortest path $p_{c_{i},c_{k_{j}}}$ between *c*_*i*_ point and every point in the set *k* is calculated. Finally, the longest path *Γ*_*C*_ of all shortest paths is chosen. This path represents the centreline of the cell *C*.

Where, *EuclideanDistance*(*C*) calculates an Euclidean distance map of every pixel of *C* to the nearest pixel outside of *C* (background pixel). $\text{ShortestPath}(M,c_{i},c_{k_{j}})$ calculates minimum cost path, using Dijkstra shortest path algorithm [37], on a graph representation of the pixels of *C* having edges weighted by *M* values.

#### *Measurements*

A digital curve (arc) or path *Γ*_*C*_ (centreline in our case) is represented by a sequence of *n* distinct pixels *p*_0_, *p*_1_,..., *p*_*n*-1_. The length element △*s*_*i*_ between two consecutive pixels centres of the digital curve *Γ*_*C*_ is △*s*_*i*_ = |*p*_*i*+1_ - *p*_*i*_|. The complete length of the digital curve which represents an open path is equal to [38]:

(15) $$L_{\left(\Gamma_{C}\right)}=L_{\left(p_{0},p_{n-1}\right)}=\overset{n-2}{\underset{i=0}{\sum }}|p_{i+1}-p_{i}|$$

**Figure 13:**
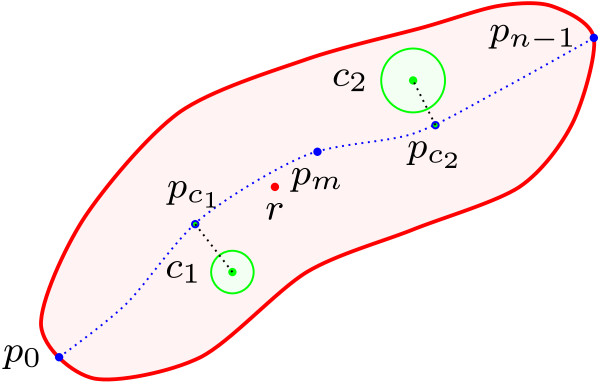
**Cell spatial measurements procedure.** A schematic diagram showing the spatial measurements taken for bacterial cells and their chemotaxis proteins.

The following measurements were implemented to determine the position of intracellular proteins relative to cell cycle (see Figure 13). 

• Cell centroid *r*,

•  cell centreline length $L_{\left(\Gamma_{C}\right)}$,

• cell area and sum of cell intensity values,

• middle point on the cell centreline *p*_*m*_,

• clusters centroids *c*_*i*_,

• point on the centreline $p_{c_{i}}$ which correspond to *c*_*i*_,

• distance form $p_{c_{i}}$ to the furthest end $\text{max}(L_{\left(p_{0},p_{c_{i}}\right)},L_{\left(p_{c_{i}},p_{n-1}\right)})$,

• distance form $p_{c_{i}}$ to the arbitrary end,

• distance form $p_{c_{i}}$ to *p*_*m*_, $L_{\left(p_{c_{i}},p_{m}\right)}$,

• cluster area and sum of cluster intensity values,

• cluster minor and major axis lengths.

## Conclusion

Presented work lays the groundwork for using DIC to produce spatiotemporal maps of proteins within the bacterial cell cycle. The use of DIC gives the potential for working with smaller levels of photobleaching and generating higher resolution maps for bacterial cell biology. This new method allows the application of high throughput analysis of low copy number bacterial proteins throughout the cell cycle. The multiple parameters measured allows the determination of how any movement or positioning varies with the cell cycle and age of cell.

Future work will be focused on high-throughput measurements of the *Rhodobacter sphaeroides* observed on temporal sequences of images. We will also investigate the applicability of the proposed concept to detect other bacterial cells observed in DIC images.

## Appendix

### Multi-dimensional Hilbert transform

The Hilbert transform of one-dimensional function *f*(*x*) is defined as follows:

(16) $$\begin{array}{c} ℋ\{f(x)\}=f_{ℋ}(x)=\frac{1}{\pi }\overset{\infty }{\underset{-\infty }{\int }}\frac{\tau }{\tau -x}\mathrm{d\tau}=f(x)*\frac{-1}{\pi x} \end{array}$$

which, in the Fourier domain, is given by:

(17) $$F_{ℋ}(u)=F(u)\cdot i\text{sign}(u)$$

where *F* and $F_{ℋ}$ are the Fourier transforms corresponding to *f* and $F_{ℋ}$ respectively. *u* represents frequency and ∗ is the convolution operator.

However, in order to use the Hilbert transform with multi-dimensional functions, a direction of reference in the Fourier domain has to be introduced [39,40]. Hence, for a direction given by a unit vector $\hat{e}$, the correspondence between a function *F* and its Hilbert transform $F_{ℋ}$ is defined as:

(18) $$F_{{ℋ}_{\hat{e}}}(u)=F(u)\cdot i{\text{sign}}_{ê}(u)$$

where **u** is a frequency coordinate and the multi-dimensional sign function is defined as:

(19) $${\text{sign}}_{ê}(u)=\left\{\begin{array}{cc} +1 & \;\;\text{if}\;\;u^{T}ê>0\\ 0 & \;\;\text{if}\;\;u^{T}ê=0\\ -1 & \;\;\text{if}\;\;u^{T}ê<0 \end{array}\right)$$

In spatial domain, this correspondence is defined by convolution between *f* and the inverse Fourier transform of $i{\text{sign}}_{ê}(u)$:

(20) $$F^{-1}\{i{\text{sign}}_{ê}(u)\}=\frac{-1}{\pi x^{T}ê}\delta_{ê}^{\text{line}}(x)$$

and we can write

(21) $$f_{{ℋ}_{ê}}(x)=f(x)*\frac{-1}{\pi x^{T}ê}\delta_{ê}^{\text{line}}(x)$$

where **x** is a spatial coordinate.

### Software

**Figure 14:**
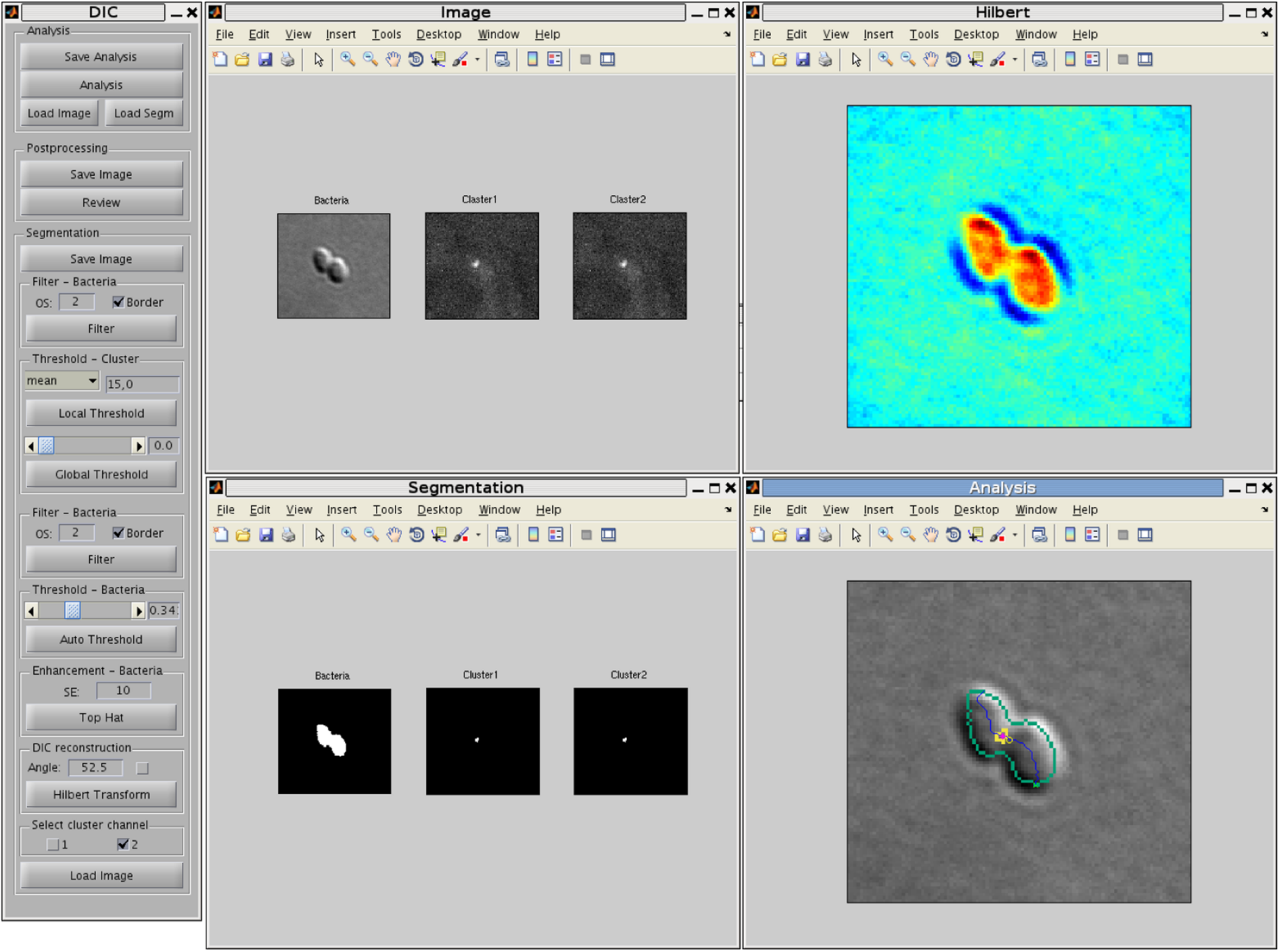
**DIC bacterial cells image analysis toolbox.** Screen-shots of the DIC bacterial cells image analysis Toolbox 1.0.

The DIC Bacterial Cells Image Analysis Toolbox (DICbc) has been implemented in MATLAB environment, see Figure 14. A detailed software description is presented below. Features: 

• Image Formats: 3 channels images -.jpg, jpeg,.png,.tiff.

• Pre-Processing Algorithms: Hilbert transform, top-hat.

• Image Thresholding: global - Otsu, local - mean, median, midgrey, Niblack, Bernsen, Sauvola.

• Image Analysis: see Image analysis Section.

• Post-Processing Algorithms: opening by reconstruction.

• Supported Platforms: UNIX/Linux, MS-Windows 2000/XP/Vista, Macintosh (OS X 10.1.4 and higher).

• Online help documentation and a test data sets.

Requirements: 

• MATLAB 6.5, R13 or higher.

• Image Processing and Bioinformatics Toolboxes of MATLAB.

• 512MB RAM.

Installation instructions for MATLAB version: 

1. Create a directory in which you would like to place the software (e.g., C: ∖∖DICbc or ∼/DICbc).

2. Move the zipped toolbox to this directory.

3. Unzip the software. This will create a directory called DICbc 1.0 which contains the Matlab code.

Running instructions: 

1. After following the steps of Installation Instructions open a session of MATLAB.

2. In the MATLAB current directory path change to the path where you install the toolbox.

3. To run DICbc type in the MATLAB command prompt: *run*.

The software is available upon request to: boguslaw.obara@durham.ac.uk

## Competing interests

The authors declare that they have no competing interests.

## Authors’ contributions

All authors participated in the design of the methods and of the related experimental methodology. All authors have read and approved the manuscript.
